# LIM and SH3 protein 2 (Lasp2) is a novel pregnane X receptor target gene in mouse liver

**DOI:** 10.1016/j.molpha.2025.100019

**Published:** 2025-02-07

**Authors:** Anja Konzack, Mikko Karpale, Tomas Smutny, Mohamed Hassanen, Piia Lassila, Maria H. Ahonen, Mahmoud-Sobhy Elkhwanky, Outi Kummu, Petr Pavek, Jukka Hakkola

**Affiliations:** 1 Research Unit of Biomedicine and Internal Medicine, Biocenter Oulu, Medical Research Center Oulu, University of Oulu and Oulu University Hospital, Oulu, Finland; 2 Department of Pharmacology and Toxicology, Faculty of Pharmacy in Hradec Kralove, Charles University, Czech Republic

**Keywords:** Liver, Nebulette, Pregnenolone-16*α*-carbonitrile, Pregnane X receptor, LASP2, Rifampicin

## Abstract

LIM and Src homology 3 (SH3) protein 2 (LASP2) is a small focal adhesion protein first identified as a splice variant of the nebulette gene (*Nebl*). As the newest member of the nebulin protein family, the regulation and function of LASP2 remain largely unknown. Our previous RNA-sequencing results identified *Nebl* as one of the most highly induced genes in the mouse liver in response to the activation of pregnane X receptor (PXR). In this study, we investigated this phenomenon further and show that PXR induces *Lasp2* instead of *Nebl*, which partially use the same exons. *Lasp2* was found to be induced in response to PXR ligand pregnenolone 16*α*-carbonitrile (PCN) treatment in mouse liver in vivo both after 4-day treatment and after long-term, 28-day treatment and in both male and female mice. Interestingly, the *Lasp2* induction was more efficient in high-fat diet–fed mice (103-fold after 4-day PCN treatment) than in the normal chow-fed mice (32-fold after 4-day PCN treatment). *Lasp2* induction was abolished in PXR knockout mice but could be rescued by re-expression of PXR, indicating that *Lasp2* induction is PXR mediated. In mouse primary hepatocytes cycloheximide did not inhibit *Lasp2* induction by PCN and a PXR binding site could be recognized upstream of the mouse *Lasp2* gene suggesting direct regulation of *Lasp2* by PXR. In human 3D hepatocytes, rifampicin induced only a modest increase in *LASP2* expression. This study shows for the first time that PXR activation strongly induces *Lasp2* expression in mouse liver and establishes *Lasp2* as a novel PXR target gene.

**Significance Statement:**

RNA-sequencing results have previously identified nebulette (*Nebl*) to be efficiently induced by pregnane X receptor activating compounds. This study shows that instead of *Nebl*, LIM and Src homology 3 (SH3) protein 2 (*Lasp2)* coding for a small focal adhesion protein and partly sharing exons with the *Nebl* gene is a novel target of pregnane X receptor in mouse liver.

## 1 Introduction

The nuclear receptor PXR (pregnane X receptor), a ligand-activated transcription factor highly expressed in the liver and intestine, has long been known for its role in regulating hepatic homeostasis and xenobiotic response. Owing to its large and flexible binding pocket, which accepts a great variety of ligands with major structural differences, PXR can sense changes in the chemical environment and adjusts cellular responses accordingly (Kliewer et al, 2002). Hence, PXR can facilitate the metabolism and detoxification of xenobiotics. Nowadays, PXR is also recognized for its roles beyond detoxification, affecting many physiological and pathological processes in the liver and intestine. PXR has been shown to be involved in energy metabolism by regulating the fate of fatty acids, lipids, and glucose. In addition, PXR has been connected to the regulation of angiogenesis, proliferation, inflammation, migration, apoptosis, and oxidative stress strongly pointing to a role in cancer growth and progression as well as in chemotherapy outcome (Pondugula et al, 2016).

**Fig. 1:**
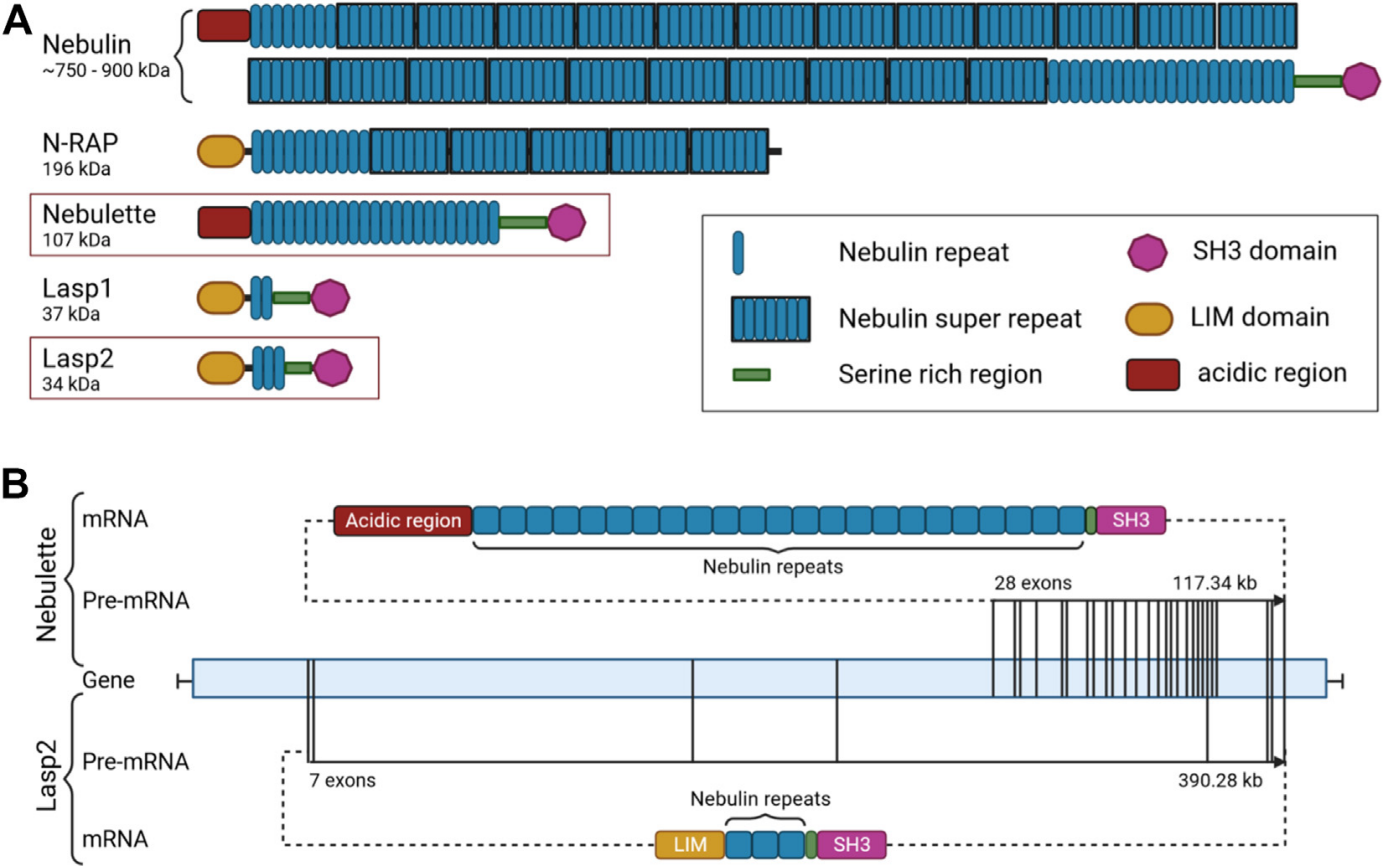
Unique features of the nebulin family of cytoskeletal proteins. (A) Schematic representation of the nebulin family members. The unifying element in all 5 proteins is the characteristic nebulin repeat, a sequence of ∼35 amino acids with a conserved actin-binding SDxxYK motif. Nebulin and N-RAP also contain groups of 7 nebulin repeats called super repeats. All but N-RAP have a serine-rich region followed by an Src homology 3 (SH3) domain in their C-terminus. In their N-terminus nebulin family members carry either a LIM domain or a unique acidic sequence. (B) Nebulette and *Lasp2* are transcribed from the same gene but from different promotors. Nebulette consists of 28 exons and *Lasp2* of 7, of which only 3 are shared between them. Although they are splicing variants of one another, they seem to have unique tissue expression patterns and functions.

The nebulin family of actin-binding proteins is composed of 5 known members (Fig. 1), namely nebulin, nebulin-related anchoring protein (N-RAP), nebulette (NEBL), LIM and Src homology 3 (SH3) protein 1 and 2 (LASP1 and LASP2), all containing the characteristic “nebulin repeat” in differing numbers as well as a unique combination of other protein domains (Pappas et al, 2011). The nebulin repeat, a ∼35-residue sequence with a highly conserved SDxxYK actin-binding motif, allows the anchoring of actin filaments to dynamic cytoskeletal structures. Indeed, nebulin, N-RAP, and NEBL are believed to bind the actin filaments of striated muscle in skeletal and heart (Pappas et al, 2011).

Unlike the other family members, NEBL and LASP2 are expressed from the same gene, but from different promoters. Although technically an isoform of NEBL, structurally, LASP2 shows extensive homology with LASP1. Functionally, LASP2 is also believed to resemble LASP1, which is thought to interact with the complex focal adhesions of fibroblasts (Grunewald et al, 2007; Pappas et al, 2011). Unlike NEBL, LASP2 expression is not limited to the cardiac muscle but is found in multiple organs, including liver, kidney, lung, colon, brain, and pancreas (Li et al, 2020). Little is known about the regulation of *Lasp2*. In the current study, we identified *Lasp2* as a novel PXR target gene in mouse liver.

## 2 Materials and methods

### 2.1 Compounds

Pregnenolone 16*α*-carbonitrile (PCN) was purchased from Abcam (ab144545), and rifampicin was a product of Sigma-Aldrich (R3501).

### 2.2 Animals

The C57BL/6N mice used in this study were obtained from the colony of the local Laboratory Animal Centre, University of Oulu. The PXR knockout (PXR-KO) mouse line was kindly provided by Dr Wen Xie, University of Pittsburgh, and has been described earlier (Xie et al, 2000). The strain was originally in the C57BL/6J background but was backcrossed at least 6 times with C57BL/6N mice to comply with the substrain of the wild-type (WT) mice used. The humanized PXR-CAR-CYP3A4/3A7 mice (Model. 11585, Taconic) used in this study have been described previously (Scheer et al, 2008).

Animals were housed on wood chip bedding with ad libitum food and water under 12-hour light/12-hour dark cycle in special pathogen-free facilities. Female mice were group housed, whereas male mice were housed in individual cages. Mice were fed regular chow (18% of calories from fat; approximate fatty acid profile [% of total fat]: 16% saturated, 23% monounsaturated, 61% polyunsaturated; Envigo td. 2018) or high-fat diet (HFD) (60% calories from fat; approximate fatty acid profile [% of total fat]: 36% saturated, 41% monounsaturated, 23% polyunsaturated; Envigo td. 06414).

All studies in animals have been carried out in accordance with the Guide for the Care and Use of Laboratory Animals as adopted and promulgated by the US National Institutes of Health and were approved by the Institution’s Animal Care and the local ethical committee (Project Authorization Board in Finland license numbers ESAVI/6357/04.10.07/2014, ESAVI/8240/04.10.07/2017, ESAVI/23252/2020).

### 2.3 PCN treatment

Animals were randomly allocated into groups and treated with PCN or vehicle for either short term (4 days) by intraperitoneal injections or long term (28 days) orally. For short-term treatment, 50 mg/kg PCN or vehicle (DMSO) in a 1:3 ratio with corn oil was injected intraperitoneally. For long-term treatment, gelatin pellets were cast in round bottom 96-well plates with blackcurrant juice. Mice were conditioned to eat the gelatin pellets for a week before starting treatment with pellets containing up to 100 mg/kg PCN for 28 consecutive days.

### 2.4 Adenoviral infection of mice

For PXR knockdown experiments, 8-week-old male C57BL/6N mice (*N* = 7/group) were transfected via tail vein with 5 × 10^9^ GFU/mL of adenovirus expressing a PXR-targeting shRNA sequence (Ad-mPXR-shRNA, Vector Biolabs, shADV-266039) or control scramble adenovirus (Ad-scr-shRNA, Vector Biolabs, 1122).

For PXR rescue experiments, 8-week-old male PXR-KO mice (*N* = 7/group) were transfected with ∼1 × 10^9^ GFU/mL of either murine PXR expressing adenovirus (Ad-GFP-mPXR, Vector BioLabs, ADV-266039) or control adenovirus (Ad-GFP, Vector BioLabs, 1060) via tail vein injection.

Four days after virus transfections, all groups were treated with 50 mg/kg PCN via intraperitoneal injection once a day for 4 days. Mice were then euthanized by CO_2_ inhalation and tissues were collected for RNA isolation.

### 2.5 Primary mouse hepatocytes

Eight-week-old C57BL/6N mice were terminally anesthetized with a medetomidine/ketamine mixture and the liver was perfused reversely from the vena cava inferior to the portal vain (with the vena cava superior tied up) using the 2-step collagenase method. After careful suspension, hepatocytes were separated by centrifugation (400 rpm, 2 minutes, room temperature) and 30,000 cells/well were plated onto 6-well plates in William’s Medium E (supplemented with 10% FBS, 20 *μ*g/L dexamethasone, 0.1% gentamycin, 1% insulin transferrin). After 6 hours, media was changed to serum-free medium, and cells were treated with 10 *μ*M or 100 *μ*M cycloheximide for 30 minutes following 24-hour treatment with 10 *μ*M PCN before being harvested for RNA isolation.

### 2.6 3D primary human hepatocyte spheroids

Cryopreserved human hepatocytes from 1 male and 3 female donors with different genetic backgrounds were purchased from BioIVT. Donor characteristics are presented in Table 1. 3D primary human hepatocyte (PHH) spheroid generation, treatment with rifampicin (10 *μ*M), real time-quantitative polymerase chain reaction (RT-qPCR), and analysis were done as previously described (Smutny et al, 2022). For RT-qPCR, a TaqMan assays (FAM) for NEBL (Hs01590549_m1, Thermo Fisher Scientific) recognizing the human splice variant 2 (LASP2) was used.

**Table 1:** PHH donor characteristics

Donor	Sex	Age (y)	Race	BMI	Post-Thaw Viability (*%*)
1	F	27	African American	28.2	96.2
2	F	30	Hispanic	30.8	94.3
3	F	39	Caucasian	35.1	97.5
4	M	58	African American	34.4	97.7

BMI, body mass index.

### 2.7 RNA extraction and qPCR

Total RNA from mouse liver samples was extracted using the RNAzol RT reagent (Sigma) according to the manufacturer's protocol. One microgram of total RNA was reverse transcribed to cDNA using random hexamer primers (Thermo Scientific) and RevertAid Reverse Transcriptase (Thermo Scientific). The cDNA was diluted 1/10 in H_2_O, and qPCR was performed using the PowerUp SYBR Green Master Mix (Thermo Scientific) and a QuantStudio 5 real-time qPCR thermal cycler (Applied Biosystems). Glyceraldehyde-3-phosphate dehydrogenase (GAPDH) and TATA-binding protein (TBP) were used as reference genes. All qPCR reactions were optimized to reach 95%–105% reaction efficiency based on primer concentration. The qPCR primers and optimized reaction concentrations are listed in Table 2. The –ΔΔCT method was used to calculate the relative mRNA fold changes where fold change = –ΔΔCT sample /-ΔΔCT control.

**Table 2:** Primer sequences for RT-qPCR

Gene	Species	Forward (5'– 3')	Reverse (5'–3')	Concentration (*nM*)
GAPDH	Mouse	GGTCATCATCTCCGCCCC	TTCTCGTGGTTCACACCCATC	100
TBP	Mouse	GAATATAATCCCAAGCGATTTG	CACACCATTTTTCCAGAACTG	300
Lasp2	Mouse	GCTGCGGAAAAGTGGTGTA	TGCTTTGGGTAGTGTGCATT	300
Nebulette	Mouse	GCCTATAAAGGGGTCCAGC	CGTCTCCGGTACTGACTTG	200
Cyp3a11	Mouse	GACAAACAAGCAGGGATGGAC	CCAAGCTGATTGCTAGGAGCA	200
PXR	Mouse	GGCTGCCTGCAGTGTTC	CTCAATAGGCAGGTCCCTAAAGTA	300
Gsta1	Mouse	TGTTGAAGAGCCATGGACAA	ATCCATGGGAGGCTTTCTCT	300
CYP3A4	Human	AAGTCGCCTCGAAGATACACA	AAGGAGAGAACACTGCTCGTG	300

### 2.8 Production of LASP2 recombinant control and immunoblotting

Lasp2 (NM_028757.3) subcloned into the pcDNA3.1(+) vector at the BamHI and EcoRI sites was ordered from Synbio Technologies, and was used to overexpress LASP2 in COS-1 cells. Cells were seeded at 3 × 10^6^ cells in 10-cm Petri dishes. The next day, at 60%–80% confluency, COS1 cells were transiently transfected with the constructed LASP2 plasmid using the jetOPTIMUS transfection kit (Polyplus), following the manufacturer’s protocol. Forty-eight hours after transfection, cells were collected and lysed using 500 *μ*L of M-PER Mammalian Protein Extraction Reagent (Thermo Scientific, 78501) supplemented with Complete, Mini, EDTA-free Protease Inhibitor Cocktail (Roche 11836170001). The cell lysate was centrifuged at 14,000*g* for 5 minutes to remove cell debris and the supernatant was collected for immunoblotting. A nontransfection control was applied.

For immunoblotting, 50 *μ*g of total liver protein or 10 *μ*g of recombinant LASP2 control were separated on a 10% polyacrylamide gel by SDS-PAGE and transferred to nitrocellulose membrane with Trans-Blot Turbo RTA Mini Nitrocellulose Transfer Kit (Bio-Rad). Membranes were blocked with Amersham ECL Prime Blocking Agent (GE Healthcare). Primary antibodies against LASP2 (Novus biologicals, NBP1-45223, 1 *μ*g/mL) and *β*-actin (Sigma, A1978, 0.5 *μ*g/mL) were applied in 0.1% TBS-Tween overnight at +4 °C. The secondary antibodies IRDye800CW donkey anti-goat (LI-COR, 926-68074, 0.2 *μ*g/mL 1:5000) and IRDye680RD goat anti-mouse (LI-COR, 926-68070, 0.04 *μ*g/mL 1:25000) in 0.1% TBS-Tween were applied for 1 hour at room temperature. Membranes were imaged on an Odyssey Fc (LI-COR) imager.

### 2.9 Analysis of public chromatin immunoprecipitation-sequencing data

The 36-nt sequence reads publicly available through NCBI (ChIP-seq BioProject ID: PRJNA239635) (Smith et al, 2014) of human hepatocyte Hu8080 cells treated with 0.01% DMSO or 10 *μ*M rifampin (Sigma) for 24 hours were aligned to the genome using Bowtie2 for single-end reads (Chipster) using default settings. Peaks were called against input using MACS2 with default settings.

### 2.10 Statistical analysis

Mouse qPCR data were log transformed before statistical analysis. Statistical significance between groups was set to be at *P* < .05 and was determined using the Student’s *t* test or one-way ANOVA with the Tukey's multiple comparisons test. Prior to analysis, normal distribution was verified using the Kolmogorov-Smirnov test (when *n* ≥ 4). n represents the number of animals or cell culture wells used. Statistical analysis was performed using GraphPad Prism 9.3.1 (GraphPad).

## 3 Results

**Fig. 2:**
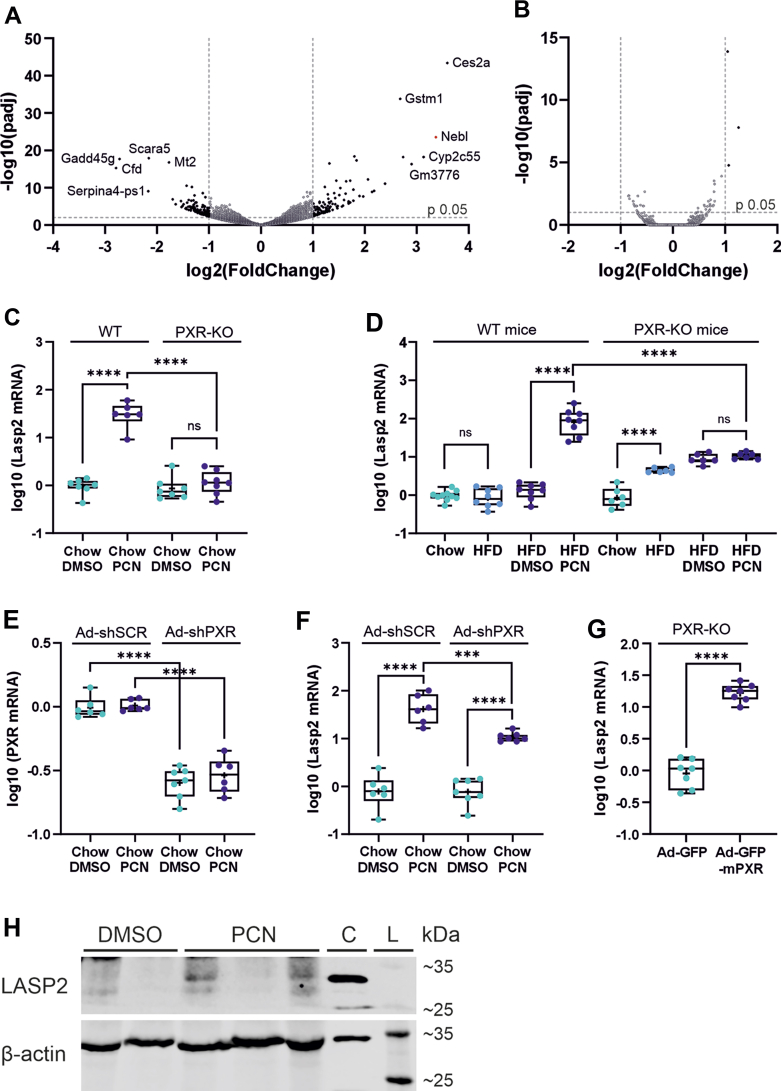
PCN induces *Lasp2* expression levels in mouse liver in a PXR-dependent manner. Volcano plots of differentially expressed genes in the livers of male HFD-fed C57BL/6N WT (A), and PXR-KO (B) mice treated with 50 mg/kg PCN or vehicle control (*n* = 3/group). The most affected genes are labeled including *Nebl* (assumed to be *Lasp2*) marked in red. (C, D) *Lasp2* expression levels in the liver following 4-day treatment with 50 mg/kg PCN or vehicle by intraperitoneal injection in male C57BL/6N mice on a chow (C) or HFD diet (D). *Pxr* (E) and *Lasp2* (F) expression in male C57BL/6N mice tail-vein injected with adenovirus carrying either shRNA against *Pxr* or the corresponding scrambled control. (G) *Lasp2* expression in male PXR-KO mice tail-vein injected with an adenovirus vector containing PXR. The boxes represent 25%–75% of log-transformed relative mRNA levels with whiskers to the smallest and largest value; +" represents the mean and the middle line the median; *n* = 6–8; ∗*P* < .05, ∗∗*P* < .01, ∗∗∗*P* < .001, ∗∗∗∗*P* < .0001. (H) Representative LASP2 immunoblot of total liver protein from male C57BL/6N mice treated with DMSO or PCN. *β*-Actin is shown to demonstrate equal loading of proteins. Panel C represents the lane of the recombinant LASP2 overexpression control and panel L represents the lane of the BlueStar Plus Prestained Protein Marker.

In a previous study, we performed RNA sequencing on the livers of HFD-fed mice treated with the prototypic rodent PXR ligand PCN, or vehicle to explore the metabolic effects of PXR activation in vivo (Karpale et al, 2021). Analysis of the RNA-sequencing data (GSE136667) not only showed an induction of cholesterol synthesis pathways by PCN, but also revealed a possible new and surprising target gene for PXR. Among the 442 differentially expressed genes (276 upregulated and 166 downregulated; adjusted *P* value < .05), nebulette (*Nebl*) was found to be the second most strongly induced gene in response to PCN treatment (Fig. 2A). Moreover, the PCN-mediated induction of nebulette appears to be PXR dependent because the effect could not be observed in RNA-sequencing data from livers of the PXR-KO mice subjected to identical treatment (GSE162196) (Karpale et al, 2023) (Fig. 2B).

Nebulette, an actin-binding protein known to be specifically expressed in the cardiac muscle, is an unlikely candidate for a PXR target gene in liver. Therefore, we hypothesized that the observed signal could represent *Lasp2*, the shorter splice variant of nebulette with a more ubiquitous expression pattern including liver (Li et al, 2020). RT-qPCR analysis with primers specific to either splice variant, *Nebl* or *Lasp2*, revealed only the presence of *Lasp2* mRNA in mice livers, whereas nebulette was undetectable (data not shown). In line with the RNA-seq data, *Lasp2* mRNA levels were found to be induced by PCN treatment in WT but not in PXR-KO male mice (Fig. 2, C and D). Knockdown of PXR in C57BL/6N male mice via adenoviral delivery of shRNA-PXR diminished the PCN-mediated induction of *Lasp2* (Fig. 2, E and F). In contrast, adenovirus-mediated re-expression of PXR was able to restore *Lasp2* induction by PCN in PXR-KO male mice (Fig. 2G), further confirming the PXR mediated induction of *Lasp2*.

*Lasp2* induction could be detected both in lean, chow-fed male mice and in obese, HFD-fed male mice (Fig. 2, C and D). However, the induction was considerably more efficient in the HFD-fed mice (103-fold) than in the chow-fed mice (32-fold). HFD alone had no effect on *Lasp2* expression in the WT mice. Interestingly, however, in the PXR-KO mice HFD caused a small 5.5-fold elevation of *Lasp2* (Fig 2D). To be noted, based on the Ct values in the qPCR measurement the *Lasp2* expression level in the control mice livers was quite low. This is also reflected in the protein levels, as no LASP2 protein could be detected from the livers of DMSO-treated control mice by immunoblotting (Fig. 2H). However, treatment with PCN resulted in detection of LASP2 protein bands in 2 of 3 tested mouse liver samples.

**Fig. 3:**
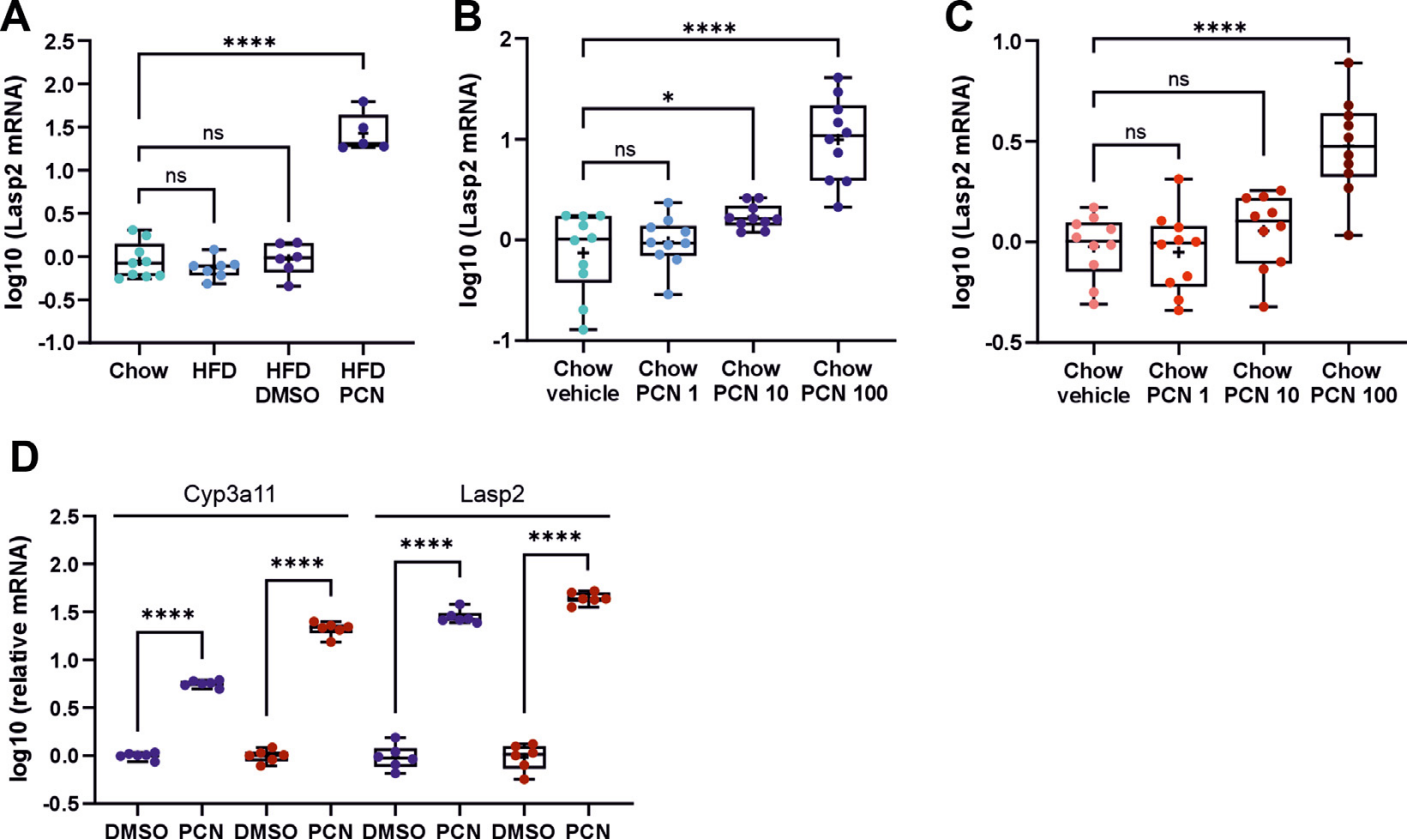
Long-term PCN treatment induces *Lasp2* expression levels in male and female mouse liver. (A) *Lasp2* expression in livers of male C57BL/6N mice on HFD diet and treated orally with 100 mg/kg PCN for 28 days. *Lasp2* mRNA levels in livers of (B) male and (C) female C57BL/6N mice treated orally with 1, 10, and 100 mg/kg PCN or vehicle for 28 days. (D) *Lasp2* expression levels in primary hepatocytes of male (blue) and female (red) C57BL/6N mice treated with DMSO or PCN (10 *μ*M) for 24 hours. The boxes represent 25%–75% of log-transformed relative mRNA levels with whiskers to the smallest and largest value; "+" represents the mean and the middle line the median; *n* = 6–8; ∗*P* < .05, ∗∗*P* < .01, ∗∗∗*P* < .001, ∗∗∗∗*P* < .0001.

*Lasp2* induction was detected also after longer term, 28-day oral PCN treatment of both HFD- and chow-fed male mice, indicating that the effect is not transient (Fig. 3, A–C). Moreover, the PCN-mediated increase in *Lasp2* mRNA levels could be observed in both sexes, although the effect was more prominent in the livers of the males compared with the females (Fig. 3, B and C). In the male mice, a statistically significant induction (1.8-fold) in *Lasp2* expression could be observed already at a PCN dose of 10 mg/kg, whereas in the female mice an induction (3.4-fold) was detected only with the highest dose of 100 mg/kg (Fig. 3, B and C). This induction pattern resembles that of *Cyp3a11* under similar conditions (Attema et al, 2024). Similar sex difference in *Lasp2* induction could not be observed in cultured primary hepatocytes isolated from male and female mice. In contrast, primary hepatocytes from a female mouse treated with 10 *μ*M PCN for 24 hours showed higher *Cyp3a11* and *Lasp2* induction compared with hepatocytes from a male mouse (Fig. 3D).

**Fig. 4:**
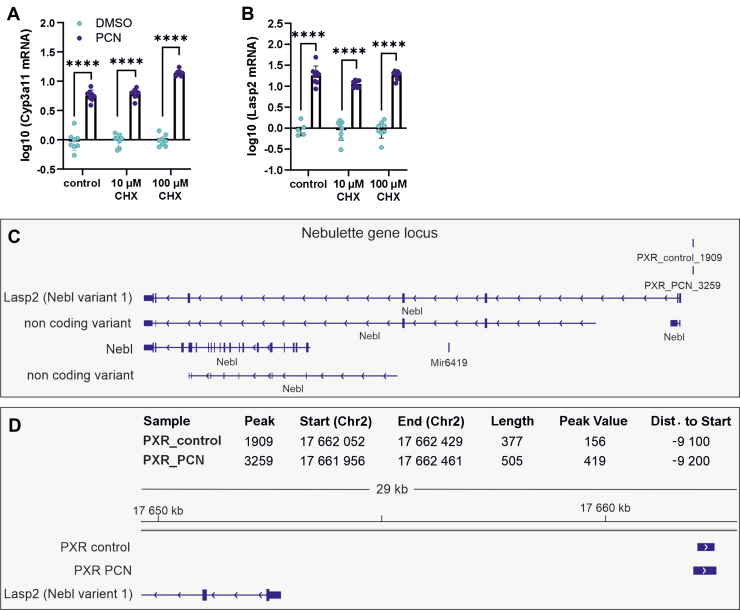
*Lasp2* as a direct target of PXR in mice. Relative mRNA levels of *Cyp3a11* (A), and *Lasp2* (B) in primary hepatocytes of C57BL/6N male mice treated with 10 *μ*M or 100 *μ*M cycloheximide (CHX) for 30 minutes following 24-hour treatment with 10 *μ*M PCN. The bars represent mean ± SD of relative mRNA levels; ∗*P* < .05, ∗∗*P* < .01, ∗∗∗*P* < .001, ∗∗∗∗*P* < .0001. (C) Nebulette locus structure and PXR binding site in control and PCN-treated mice according to the ChIP-sequencing data published by Cui et al (2010). (D) Closeup to the PXR binding site upstream of *Lasp2* gene in control and PCN-treated mice. The numbering is based on mm9 alignment.

To elucidate whether *Lasp2* is a direct target of PXR, primary mouse hepatocytes of WT male mice were treated with cycloheximide to inhibit protein synthesis prior to the activation of PXR with PCN. Treatment with cycloheximide neither inhibited the induction of *Lasp2* nor that of *Cyp3a11*, a well known PXR target, suggesting direct regulation of *Lasp2* by PXR (Fig. 4, A and B).

In the next step, we did data mining of previously published PXR chromatin immunoprecipitation (ChIP)-sequencing data performed in PCN-treated mice liver by Cui et al (2010). Analysis of these data revealed the presence of a PXR binding site approximately 9 kb upstream of the *Lasp2* gene in both PCN and vehicle-treated mouse livers (Fig. 4, C and D). Moreover, PXR-DNA binding at this site was increased by 2.22-fold (PCN/corn oil) upon PCN treatment, whereas no change in *Nebl* mRNA expression was detected by Cui et al (2010). The ChIP-sequencing data thus indicate direct binding of PXR to the putative regulatory region of *Lasp2* strongly supporting the concept that it is a direct target gene of PXR.

**Fig. 5:**
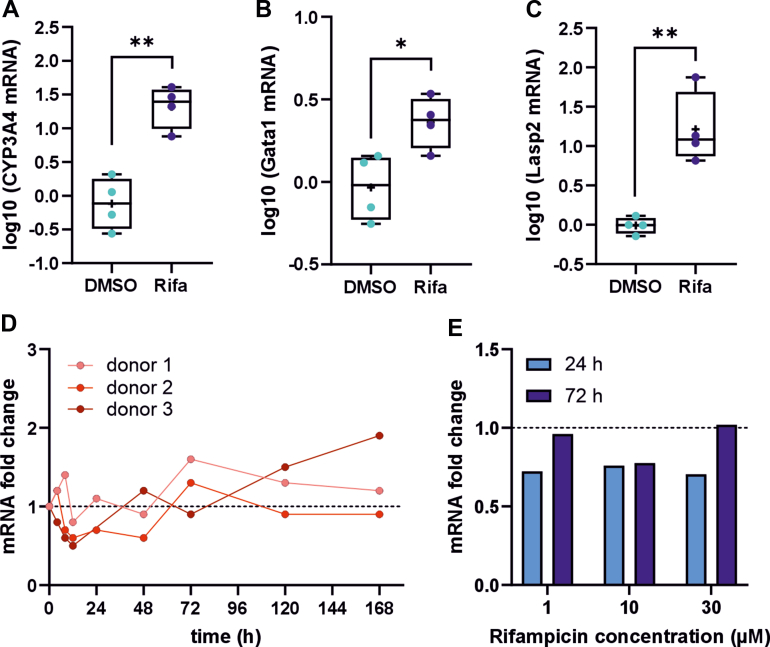
*Lasp2* induction by PXR activation in humanized and human models. Relative mRNA levels of *CYP3A4* (A), *Gsta1* (B), and *Lasp2* (C) in humanized PXR-CAR-CYP3A4/3A7 male mice treated with 4 doses of 10 mg/kg rifampicin or vehicle (DMSO) on consecutive days. The boxes represent 25%–75% of log-transformed relative mRNA levels with whiskers to the smallest and largest value; "+" represents the mean and the middle line the median; *n* = 6–8; ∗*P* < .05, ∗∗*P* < .01, ∗∗∗*P* < .001, ∗∗∗∗*P* < .0001. 3D PHHs from (D) 3 female donors and (E) 1 male donor treated with rifampicin or DMSO for indicated time points. The rifampicin concentration in the cultures from the female donors was 10 *μ*M. mRNA expression of *LASP2* was analyzed by RT-qPCR. Data were normalized to the *TBP* reference gene and presented as the fold change expression relative to the vehicle control at the same time point, which was set to be 1.

We also investigated whether the PXR-mediated *LASP2* induction could occur in human liver. The ligand-binding domain of PXR displays major species differences in ligand preference. Although PCN is a poor ligand for human PXR, rifampicin efficiently induces the human PXR but has little effect on the murine PXR. In contrast to the ligand-binding domain, the DNA-binding domain of PXR is rather well conserved between species and therefore the PXR-regulated genes and functions display substantial overlap between humans and mice. As a first step, we evaluated whether the human PXR is able to regulate *Lasp2* gene in a humanized mouse. Four-day treatment of PXR-CAR-CYP3A4/3A7 humanized male mice with the human PXR-specific ligand rifampicin (10 mg/kg) resulted in a 25-fold induction of human *CYP3A4* (Fig. 5A), a 2.4-fold induction of murine glutathione S-transferase A1 (*Gsta1*) (Fig. 5B), and a 26-fold induction of *Lasp2* (Fig. 5C). Thus, *Lasp2* was induced by rifampicin as efficiently as the prototypic PXR target gene *CYP3A4* and 10 times higher than the well known target *Gsta1,* indicating that human PXR is able to regulate *Lasp2* gene in the context of mouse genome. To further investigate if human *LASP2* is regulated by PXR, we treated 3D PHH spheroids with rifampicin and investigated *LASP2* mRNA expression at different time points (Fig. 5D). In 2 of the 3 donors, a very modest *LASP2* induction was observed at late time points (after 72 hours) (Fig. 5D). All the 3 donors were female. To test the possibility that there could be some sex differences in the human induction of LASP2 in response to PXR activation, we performed an additional 3D spheroid culture experiment with hepatocytes from a male donor. The spheroids were treated with 3 different concentrations of rifampicin (1, 10, or 30 *μ*M), and *LASP2* mRNA was measured 24 and 72 hours after treatments. No effect on *LASP2* mRNA expression was observed in any of the concentrations or either time point (Fig. 5E). Finally, we analyzed publicly available ChIP-sequencing data on PXR binding sites performed in rifampicin-treated human hepatocytes (Smith et al, 2014). No PXR binding sites near the *NEBL* gene could be identified.

## 4 Discussion

In this study, we show for the first time that PXR activation induces *Lasp2* expression in mouse liver. In many experimental settings, *Lasp2* was found to be among the most highly induced genes in response to PXR ligand. Furthermore, the *Lasp2* induction was detected in both short- and long-term treatments. It should, however, be kept in mind that, based on the Ct values in the qPCR measurements, the basal expression level in the controls was rather low. We could detect LASP2 protein in the HFD-fed, PCN-treated mice but not in the vehicle-treated mice also supporting the idea that the constitutive expression level of LASP2 in the liver is low. Using several different KO, knockdown, and overexpression methods, we showed that *Lasp2* induction is indeed PXR mediated. Furthermore, *Lasp2* appears to be a direct target gene of PXR and there is a ligand responsive PXR binding site upstream of the nebulette locus. Thus, *Lasp2* expression appears to be controlled by chemical exposure.

Although our study well characterizes a robust induction of *Lasp2* in mouse liver, we were able to provide very limited evidence on *LASP2* regulation by PXR in humans. In PXR-CAR-CYP3A4/3A7 humanized mice *Lasp2* was efficiently induced by rifampicin; however, this still represents a mouse genome response. In 3D PHH spheroids, a modest rifampicin-mediated induction of *LASP2* could be observed in 2 of 3 female donors and no induction was detected in the male donor. Compared with the previously observed responses of classical target genes of PXR, such as *CYP3A4*, in a similar setting, the observed *LASP2* induction in the female donor hepatocytes was small and delayed (Smutny et al, 2022). These results suggest that there are important species differences in *LASP2* regulation and that the human *LASP2* is much less responsive to PXR activation in liver than the mouse gene. Whether human *LASP2* induction by PXR agonists could take place in vivo or could be detected in different experimental conditions still requires future confirmation. Moreover, the intestine could also represent a potentially relevant tissue for PXR-mediated *LASP2* regulation as both proteins are expressed there as well.

So far, only little is known about the functional role of LASP2. Interestingly, we observed that *Lasp2* induction by PCN was more efficient in HFD-fed than normal chow-fed mice, which could be speculated to hint to some role in metabolism. Several studies have linked LASP2 to cancer development, with mounting evidence indicating a tumor suppressive role (Pappas et al, 2011; Wang et al, 2017; Yang et al, 2018; Li et al, 2020). LASP2 was found to be downregulated in several human cancers, including hepatocellular carcinoma (Li et al, 2020), colorectal cancer (Wang et al, 2017), nasopharyngeal carcinoma (Yang et al, 2020), and bladder cancer (Yang et al*,* 2018). In line, knockdown of *LASP2* enhanced the malignant phenotype of human liver (Li et al, 2020) and colorectal cancer (Wang et al, 2017) cells, whereas overexpression was found to inhibit proliferation and migration in human colorectal (Wang et al, 2017), bladder (Yang et al, 2018), pancreatic (Zhang et al, 2019), and liver (Li et al, 2020) cancer cell lines.

PXR has also been reported to be differentially expressed in various types of cancer. In the liver, recent studies point toward a tumor suppressive role for PXR. The expression levels of PXR and its target genes were found to be downregulated in diethylnitrosamine-induced hepatocellular carcinoma in C57BL/6J mouse (Kotiya et al, 2016). In line with this, PXR deficiency promoted diethylnitrosamine-induced hepatic cancer and decreased survival in PXR-KO mice (Shi et al, 2024), whereas overexpression of PXR was shown to diminish the malignancy of human cancer cell lines by reducing cell migration, adhesion, and invasion (Kotiya et al, 2016). Thus, in theory the induction of *Lasp2* by PXR activation could be a tumor protective strategy in response to chemical exposure.

## Conflict of interest

The authors declare no conflicts of interest.
